# Structure and Function of Honeybee α-Amylase of Glycoside Hydrolase Family 13 Subfamily 15

**DOI:** 10.3390/molecules31152615

**Published:** 2026-07-27

**Authors:** Wataru Saburi, Yushi Takahashi, Shiho Takei, Toyoyuki Ose, Haruhide Mori

**Affiliations:** 1 Research Faculty of Agriculture, Hokkaido University, Kita 9, Nishi 9, Sapporo 060-8589, Hokkaido, Japan; hmori@agr.hokudai.ac.jp; 2 Japan Food Research Laboratories, Bunkyo, Chitose 066-0052, Hokkaido, Japan; takahashiyu@jfrl.or.jp; 3 Faculty of Advanced Life Science, Hokkaido University, Kita 10, Nishi 8, Sapporo 060-0810, Hokkaido, Japan; takei@castor.sci.hokudai.ac.jp (S.T.); ose.toyoyuki@sci.hokudai.ac.jp (T.O.)

**Keywords:** α-amylase, honeybee, honey, GH13_15, starch granules, subsite map

## Abstract

α-Amylase, ubiquitously distributed across diverse organisms, catalyzes the hydrolysis of the internal α-(1→4)-linkage of α-(1→4)-glucan including starch as an essential energy source. Insect α-amylases, which belong to the glycoside hydrolase family 13 subfamily 15 (GH13_15), are important for optimal larval growth and adult longevity. Honeybee (*Apis mellifera*) α-amylase (AMA) is expressed in the hypopharyngeal glands of forager bees and secreted into honey. AMA in honey, which is important for food quality control, has been partly characterized. However, its structure–function relationship is poorly understood. Herein, we present biochemical, structural, and mutational analyses of AMA. Kinetic analysis using *p*-nitrophenyl maltooligosaccharides and their 4,6-benzylidene-modified derivatives revealed a subsite affinity map of AMA. AMA contains high-affinity subsites −3, −2, +1, and +2, similar to those of the mammalian α-amylases of GH13_24. Most AMA substrate-binding residues are conserved in the GH13_24 enzymes. Mutational analysis revealed that Leu175 is crucial at subsites −3/−2 for reactions with oligo- and polysaccharides. Furthermore, Trp77 at subsites −3/−2 and Lys210 at subsite +2 are suggested to be involved in the proper binding of long-chain substrates. AMA shares a surface sugar-binding site with mammalian α-amylases, where Asn281, Trp285, and Trp293 are essential for the binding and degradation of starch granules.

## 1. Introduction

α-Amylase (EC 3.2.1.1) is a glycoside hydrolase (GH) that hydrolyzes the internal α-(1→4)-linkage of α-(1→4)-glucan in starch, glycogen, and related poly- and oligosaccharides with net retention of an anomeric configuration of substrates [1]. This enzyme is distributed across a wide variety of living organisms, including microorganisms, plants, and animals, and plays a central role in breaking down α-(1→4)-glucan as an essential energy source. According to the sequence-based classification of GHs in the CAZy database (https://www.cazy.org) [2], α-amylase is included in GH families 13, 57, 119, and 126 (GH13, GH57, GH119, and GH126, respectively). GH13 includes α-amylases from all kingdoms of organisms, while GH57 includes α-amylases of archaeal and bacterial origins and GH119 and GH126 include bacterial α-amylases.

GH13 is one of the largest GH families and is composed of various kinds of enzymes acting on α-glucans: hydrolases such as α-amylase, α-glucosidase (EC 3.2.1.20), neopullulanase (EC 3.2.1.135), and limit dextrinase (EC 3.2.1.41); glycosyltransferases such as amylosucrase (EC 2.4.1.4), starch branching enzyme (EC 2.4.1.18), and cyclodextrin glucanotransferase (EC 2.4.1.19); and phosphorylases such as sucrose phosphorylase (EC 2.4.1.7) and α1,4-glucan:maltose-1-P maltosyltransferase (EC 2.4.99.16). These enzymes share three domains: domain A, a catalytic (β/α)_8_-barrel; domain B, a long loop connecting the third β-strand and α-helix of domain A (β→α loop 3); and domain C, an antiparallel β-strand domain following domain A [3]. The substrate-binding site is formed at the interface between domains A and B. The catalytic nucleophile Asp and general acid/base Glu for the double-displacement mechanism are located at the C-termini of β→α loops 4 and 5, respectively. Additionally, the Asp at the C-terminus of β→α loop 7 facilitates distortion of the d-glucosyl residue at subsite −1 and stabilizes an oxocarbenium ion-like transition state as the second catalytic Asp. GH13 enzymes are divided into 49 subfamilies based on sequence similarity, and most subfamilies appear monofunctional [4,5]. α-Amylases from fungi belong to GH13 subfamily 1 (GH13_1); those from plants, GH13_6; those from animals, GH13_15 and GH13_24; and those from bacteria, GH13_5, 19, 21, 27, 28, 32, 36, 37, 39, 41, 42, 43, 45, 46, 47, 48, and 49. GH13_7 and GH13_20 contain bacterial and archaeal α-amylases.

Insect α-amylases of GH13_15 play an essential role in obtaining energy for optimal larval growth and adult longevity [6]. Various α-amylases were identified in pests and economically important insects, and their enzymatic functions have been investigated [7]. The honeybee (*Apis mellifera*) possesses a single gene encoding α-amylase [8], which is expressed in the hypopharyngeal glands of forager bees [9]. As this enzyme is secreted into honey [10,11,12], it degrades starch included in food containing honey as an ingredient, causing viscosity loss [13]. Honey α-amylase is used as a key indicator for distinguishing genuine from counterfeit honey including foreign amylolytic enzymes [14]. Although α-amylase is important for honeybee growth and food quality, the structure–function relationship of honeybee α-amylases (AMA) is scarcely understood. In this study, to deepen our understanding of the functions and structure of AMA, we identified honey α-amylase as AMA and described its biochemical functions, its crystal structure in complex with a pseudomaltooligosaccharide, and the functions of its substrate-binding residues.

## 2. Results

### 2.1. Identification of Honey α-Amylase and Production of Recombinant AMA

**Figure 1:**
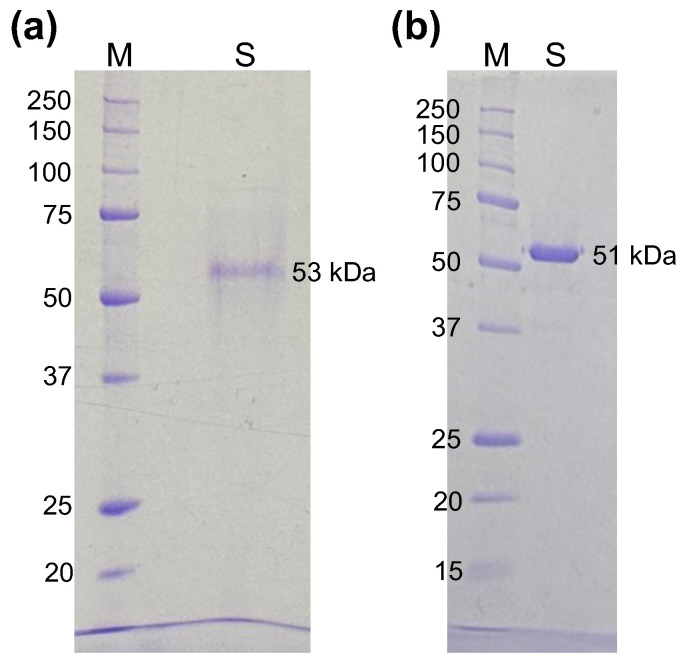
SDS-PAGE analysis of purified AMA from honey (**a**) and culture supernatant of *P. pastoris* transformant (**b**). Lane M and S indicate molecular size marker and purified sample (1 μg), respectively.

As α-amylase in honey was not confirmed to be identical to AMA, the α-amylase was purified from 50 g of commercial honey using anion-exchange and hydrophobic interaction column chromatography. The starting material (diluted and dialyzed honey) contained 3.24 U of α-amylase; 0.890 U of the purified protein (5.86 U/mg) was obtained (Figure 1a). Using the specific activity of the purified enzyme, the amount of α-amylase in honey was estimated at 0.0111 mg/g honey, consistent with the previous study (0.0136 mg/g honey [11]). A protein band of 53 kDa, which corresponds to a previously purified honey α-amylase [10,11,12], was confirmed as AMA (NCBI reference sequence number, NP_001011598.1) using liquid chromatography–tandem mass spectrometry (LC–MS/MS) analysis of tryptic peptides, whereby the resulting spectra matched the deduced sequence with 77% coverage. Met1–Gly17 was predicted to be a signal peptide with a probability of 0.980 using SignalP 6.0 [15]. The theoretical molecular mass of the mature protein is 54,253.94 Da. Two possible *N*-glycosylation sites (Asn228-Arg229-Thr230 and Asn408-Gly409-Ser410) are present in the mature AMA sequence.

As the production of mature AMA in *Escherichia coli* SolBL21 was unsuccessful (the recombinant protein was produced in insoluble form), a recombinant protein with an α-factor signal sequence instead of the original signal peptide was extracellularly produced in *Komagataella phaffii* (*Pichia pastoris*). From 0.8 L of the culture broth after 96 h of cultivation, recombinant AMA was purified to homogeneity by utilizing β-cyclodextrin (CD) affinity and gel filtration column chromatography (Figure 1b), obtaining 14.4 mg of purified enzyme with a specific activity of 96.9 U/mg toward 2 mM *p*-nitrophenyl 4,6-benzylidene-α-maltoheptaoside (blocked pNPG7). The purified enzyme showed a single 51 kDa band on an SDS-PAGE gel. The molecular mass determined by the SDS-PAGE analysis was slightly lower than the theoretical mass. The expression system of *P. pastoris* was used for all of the AMA mutants as described later.

### 2.2. Enzymatic Characterization of AMA

#### 2.2.1. Effects of pH and Temperature on Activity and Stability

**Figure 2:**
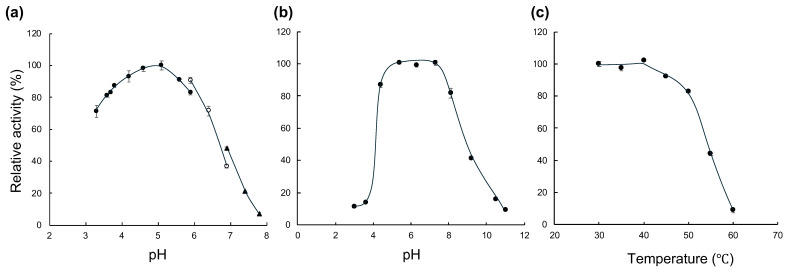
Effects of pH and temperature on activity and stability of AMA. (**a**) pH activity profile. Black circles, white circles, and black triangles are reactions in sodium acetate, MES-NaOH, and HEPES-NaOH, respectively. (**b**) pH stability (4 °C, 24 h). (**c**) Temperature stability (pH 5.0, 15 min). Values and error bars are average and standard deviation for three independent experimental values.

AMA showed the highest activity toward soluble starch at pH 5.1 (Figure 2a). It was stable between pH 5.4 and 7.3 (4 °C, 24 h) and at ≤40 °C (pH 5.0, 15 min) (Figure 2b,c). These properties are consistent with those of honey α-amylase: optimum pH, pH 4–6; stable pH range, pH 4–6 (4 °C, 24 h); and ≤40 °C (pH 6.0, 60 min) [12].

#### 2.2.2. Effects of Calcium and Chloride Ions on Activity and Stability

**Figure 3:**
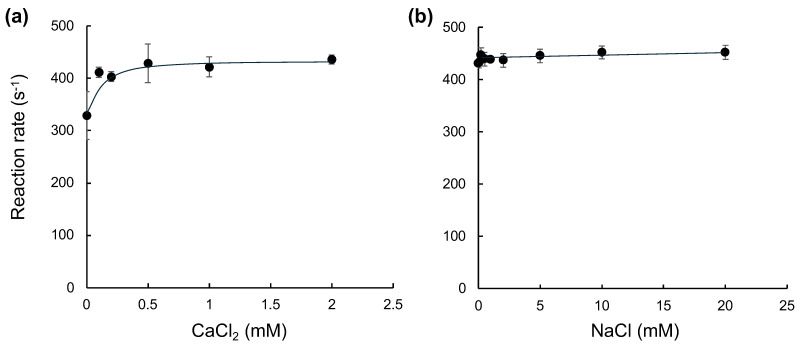
Effects of CaCl_2_ and NaCl on the activity of AMA. (**a**) CaCl_2_ and (**b**) NaCl. Values and error bars are average and standard deviation for three independent experimental values.

The reaction rate of AMA with soluble starch was measured in the presence of various concentrations of CaCl_2_ and NaCl (Figure 3). The reaction rate of AMA slightly increased with increasing CaCl_2_ concentration up to 0.5 mM, whereas it was unchanged by the addition of NaCl. The thermal denaturation of AMA in 10 mM HEPES-NaOH (pH 7.0) was monitored in the presence and absence of 2 mM CaCl_2_ or 2 mM EDTA-2Na using differential scanning calorimetry (DSC, Table 1). A non-two-state model fitted the experimental values well (Δ*H*_cal_/Δ*H*_HV_ > 1). Δ*H*_cal_ was not significantly different between the sample without additives (1050 kJ/mol) and that with EDTA-2Na (972 kJ/mol), but was 1.3-fold higher in the presence of CaCl_2_ (1390 kJ/mol). The *T*_m_ value was 68.3 °C in the presence of CaCl_2_, while it was 55.6 °C and 55.9 °C without additives and with EDTA-2Na, respectively.

#### 2.2.3. Kinetic Analysis of Reactions with Oligo- and Polysaccharides

**Figure 4:**
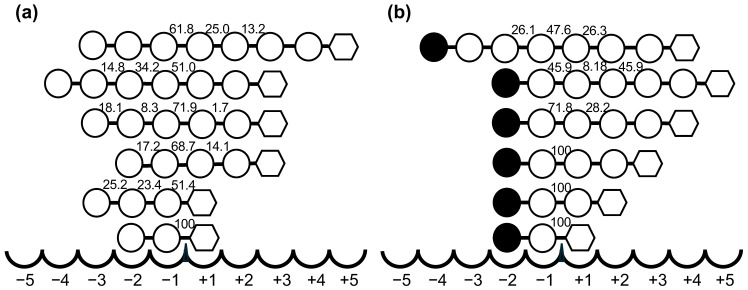
Bond cleavage frequency of AMA. (**a**) Unmodified pNP-maltooligosaccharides. (**b**) Blocked pNP-maltooligosaccharides. Most frequent binding mode is shown. Numbers above linkage indicate bond cleavage frequency. White and black circles are d-glucosyl and 4,6-benzylidene d-glucosyl residues, respectively. Hexagons indicate *p*-nitrophenyl group.

The bond cleavage frequency (BCF) of AMA for 1 mM non-modified and blocked *p*-nitrophenyl (pNP)-maltooligosaccharides (degree of polymerization [DP]: 2–7) was determined (Figure 4a and Figure S1). For unmodified pNP-maltooligosaccharides, AMA cleaved the second to fourth linkages from the non-reducing end of pNPG6 and the third to fifth linkages from the non-reducing end of pNPG7, while generating products with a decrease in DP by one from pNPG3–5. These products may be generated through transglycosylation and the subsequent rapid hydrolysis of the resulting intermediate, although such transglycosylation products were not directly detected. For blocked pNPG2–5, which does not serve as glycosyl acceptors for 4-α-transglycosylation, AMA cleaved the second linkage from the non-reducing end (Figure 4b). The linkage at the non-reducing end of these substrates was not hydrolyzed. The cleaved linkages in the blocked pNPG6 and 7 were consistent with those in the corresponding unmodified substrates, but their BCF values differed. For blocked pNPG6, the BCF for the second linkage from the non-reducing end was higher, and that for the third linkage was lower than that for pNPG6. For blocked pNPG7, the BCF for the fourth and fifth linkages from the non-reducing end was higher, and that for the third linkage was lower than that for pNPG7.

**Figure 5:**
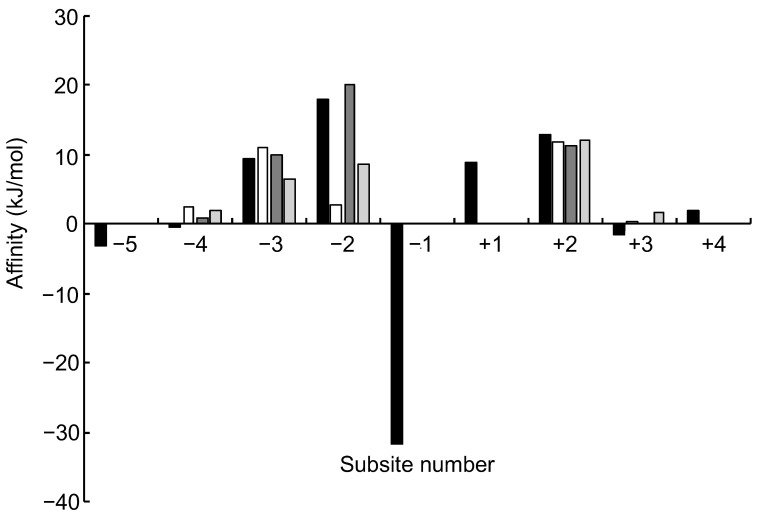
Subsite affinity maps of AMA and related enzymes. Black bar, AMA; white bar, Colorado potato beetle α-amylase [18]; dark gray bar, human salivary α-amylase [18]; and light gray bar, porcine pancreatic α-amylase [18]. Affinity for subsites −1 and +1 of the compared enzymes were not determined.

The subsite affinity (*A*_n_; n, DP) of AMA was estimated from the BCF for the respective productive binding modes *p*_n_,_j_ (j, subsite number occupied by the reducing-end d-glucosyl residue) and *k*_cat_/*K*_m_ for non-modified and blocked pNP-maltooligosaccharides (Figure 5). The *k*_cat_/*K*_m_ of AMA significantly increased with increasing substrate DP, up to DP6 (Table 2). *A*_+1_ and *A*_+2_ were calculated from *k*_cat_/*K*_m_ values for the blocked pNP-maltooligosaccharides. The others were calculated from the *k*_cat_/*K*_m_ and BCF values for the unmodified substrates as follows:

Subsite +1: *A*_+1_ = RTln{(*k*_cat_/*K*_m_)_blocked pNPG3_/(*k*_cat_/*K*_m_)_blocked pNPG2_} = 8.80 kJ/mol

Subsite +2: *A*_+2_ = RTln{(*k*_cat_/*K*_m_)_blocked pNPG4_/(*k*_cat_/*K*_m_)_blocked pNPG3_} = 12.8 kJ/mol

Subsite +3: *A*_+3_ = RTln{*p*_7,3_(*k*_cat_/*K*_m_)_pNPG7_/*p*_6,2_(*k*_cat_/*K*_m_)_pNPG6_} = −1.62 kJ/mol

Subsite +4: *A*_+4_ = RTln{*p*_7,4_(*k*_cat_/*K*_m_)_pNPG7_/*p*_6,3_(*k*_cat_/*K*_m_)_pNPG6_} = 1.74 kJ/mol

Subsite −2: *A*_−2_ = RTln{(*k*_cat_/*K*_m_)_pNPG2_/(*k*_cat_/*K*_m_)_pNPG_} = 17.8 kJ/mol

Subsite −3: *A*_−3_ = RTln{*p*_3,0_(*k*_cat_/*K*_m_)_pNPG3_/(*k*_cat_/*K*_m_)_pNPG2_} = 9.30 kJ/mol

Subsite −4: *A*_−4_ = RTln{*p*_7,3_(*k*_cat_/*K*_m_)_pNPG7_/*p*_6_,_3_(*k*_cat_/*K*_m_)_pNPG6_} = −0.593 kJ/mol

Subsite −5: *A*_−5_ = RTln{*p*_7,2_(*k*_cat_/*K*_m_)_pNPG7_/*p*_6_,_2_(*k*_cat_/*K*_m_)_pNPG6_} = −3.27 kJ/mol

*A*_−1_ was estimated from the *k*_cat_/*K*_m_ and BCF for pNPG6 and pNPG7 to be −31.6 kJ/mol according to the subsite theory [16,17]:

*B*_n,j_ = Σ*A*_n_ = RTln{*p*_n_,_j_(*k*_cat_/*K*_m_)_pNPGn_/0.018*k*_int_}

In this estimation, the *k*_cat_ for amylose EX-1 (average DP17) was considered as *k*_int_. The *k*_cat_/*K*_m_ values of AMA for pNPG6 and pNPG7, calculated using the subsite affinity, were 7340 and 9180 s^−1^mM^−1^, respectively, which were in good agreement with the experimental values (Table 2). AMA possesses high-affinity subsites at −3, −2, +1, and +2.

### 2.3. Crystal Structure of AMA in Complex with an Acarbose-Derived Oligosaccharide

**Figure 6:**
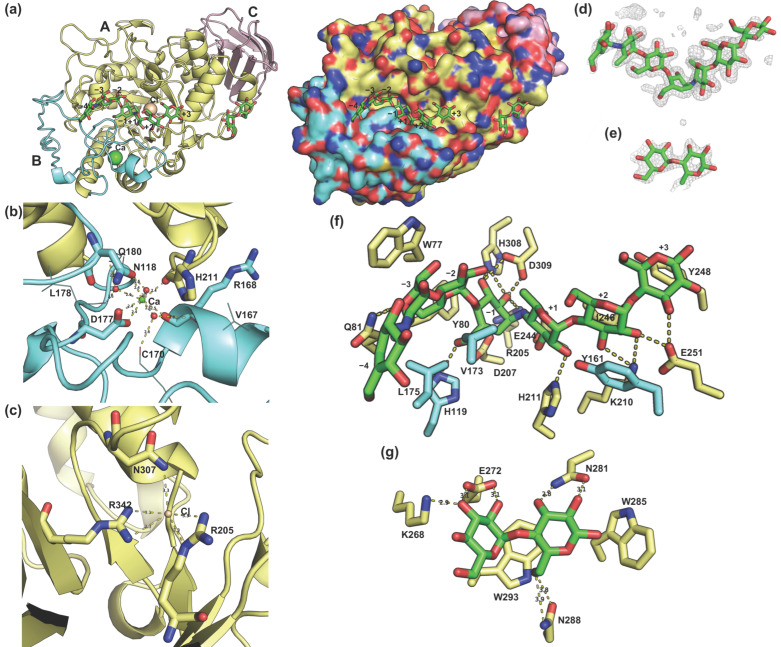
Crystal structure of AMA. (**a**) Overall structure. Domains A, B, and C are shown in yellow, light blue, and pink, respectively. (**b**) Calcium ion binding site. Red spheres indicate water molecules. Residues, forming hydrogen bonds with water molecules, are shown in thin lines, and residues coordinating calcium ion are shown in stick representation. (**c**) Chloride ion binding site. (**d**,**e**) Polder maps of the acarbose-derived heptasaccharide (**d**) and maltose (**e**) (contoured at 2.5 σ). (**f**) Active site. (**g**) Maltose-binding site. Sugars are shown in green sticks. Yellow dashed lines indicate possible hydrogen bonds (distance of two atoms, 2.7–3.3 Å) except Asn288. Distance (Å) between two the atoms connected by dashed line is shown on the dashed line.

AMA crystal was prepared by co-crystallization with a pseudomaltotetraose acarbose. The structure of AMA in complex with an acarbose-derived heptasaccharide was solved at 1.9 Å resolution (Figure 6, Table 3). The heptasaccharide was produced by transglycosylation; the non-reducing-end trisaccharide moiety of acarbose was transferred to the valienamine residue of another acarbose molecule. AMA is composed of three canonical GH13 domains: A, B, and C (Figure 6a). Its root-mean-square deviations from structurally characterized GH13_15 enzymes, *Drosophila melanogaster* α-amylase (DMA) (PDB ID: 8ORP [19]) and *Tenebrio molitor* α-amylase (TMA) (PDB ID: 1JAE [20]), are 0.427 Å and 0.570 Å, respectively. AMA and TMA lack the flexible long β→α loop 7 found in DMA [19]. Electron density was observed for an *N*-acetyl-d-glucosaminyl moiety bound to the Asn228 side chain, which is part of the *N*-glycosylation motif (Asn228-Arg229-Thr230). Calcium and chloride ions are bound to the corresponding sites in the GH13_15 enzymes (Figure 6b,c). Electron density corresponding to the acarbose-derived heptasaccharide and a maltose was observed in the cleft formed at the interface of domains A and B and on the long β→α loop 6, respectively (Figure 6d–g).

The acarbose-derived heptasaccharide covers subsite −4 to +3, as observed in DMA [19] (Figure 6f). The members of the catalytic triad, Asp207, Glu244, and Asp309, are located at positions suitable for serving as catalytic nucleophile, general acid/base catalyst, and transition state stabilizer, respectively. All hydroxy groups of the valienamine moiety at subsite −1 form a hydrogen bond with His119, Arg205, His308, and Asp309; Tyr80 stacks against this moiety, as observed in most GH13 enzymes [3]. Trp77 is located at an intermediate position between the 6-deoxy-α-d-glucosyl and α-d-glucosyl residues at subsites −3 and −2, respectively, forming an aromatic platform for the β-face of these sugar residues. Val173 and Leu175 are also located at intermediate positions between subsites −3 and −2, but make hydrophobic contacts with the α-face of the sugar residues. Gln81 is positioned to form hydrogen bonds with O6 of α-d-glucosyl residue at subsite −2 and O5 of 6-deoxy-α-d-glucosyl residue at subsite −3. At the +1 subsite, His211 and Glu244 are oriented to form hydrogen bonds with O2 and O3 of the 6-deoxy-α-d-glucosyl residue, respectively. At the +2 subsite, Tyr161 stacks against the α-d-glucosyl residue from the β-face, and Ile246 makes hydrophobic contact with the same residue from the α-face. Lys210 forms hydrogen bonds with O2 and O3. Glu251 is suitable to form hydrogen bonds with O2 of the α-d-glucosyl residue at subsite +2 and O3 of the d-glucose residue at subsite +3. Tyr248 presumably makes hydrophobic contact with the d-glucose residue from the α-face at subsite +3. No residues interacting with the valienamine unit at subsite −4 were identified.

For maltose binding to the β→α loop 6 of domain A, Trp293 is located at an intermediate position between two sugar residues and forms an aromatic platform (Figure 6g). O2 and O3 of the reducing-end d-glucose residue of maltose can form hydrogen bonds with Asn281. Lys268 and Glu272 can form hydrogen bonds with O2 and O3 in the non-reducing-end d-glucosyl residue.

### 2.4. Functional Analysis of the Substrate-Binding Residues Through Site-Directed Mutagenesis

#### 2.4.1. Substrate-Binding Residues at Subsites −3, −2, +2, and +3

**Figure 7:**
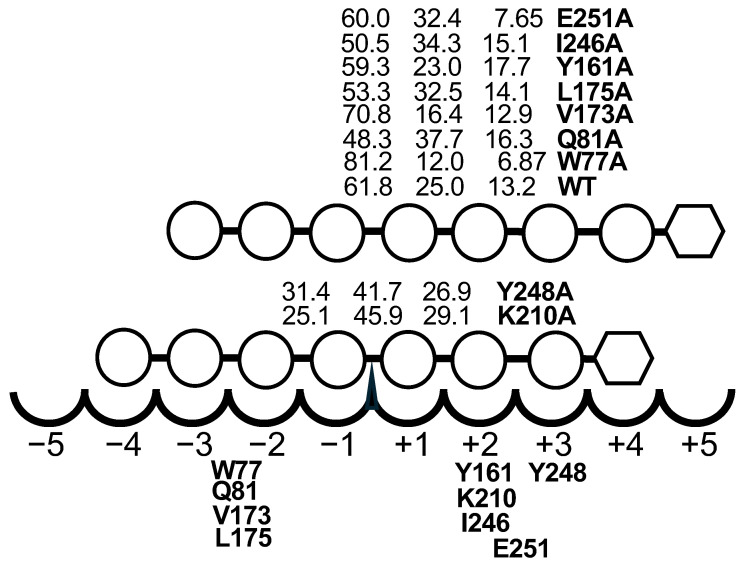
Bond cleavage frequency of AMA mutants for pNPG7. Most frequent binding mode is shown. Numbers above linkage indicate bond cleavage frequency. White circles and hexagons indicate d-glucosyl residue and pNP group, respectively.

To characterize the residues forming subsites −3, −2, +2, and +3, Ala was substituted at these sites. The Q81A and L175A mutations reduced the activity toward 2 mM blocked pNPG7 to 59.2 and 39.3% of the wild type (WT) activity, respectively, whereas the other mutations increased the activity by 1.9 to 9.3-fold (Table 4). Notably, Y161A, I246A, and E251A exhibited high activities, which were 9.3-, 3.4-, and 4.1-fold higher than that of the WT, respectively. The BCFs of the AMA mutants toward pNPG7 were analyzed (Figure 7). Among the analyzed mutants, K210A and Y248A produced significantly more pNPG2 and pNPG3, and less pNPG4 than the WT. These mutants produced pNPG3 as their most abundant product, whereas the WT predominantly produced pNPG4. The W77A mutant increased the release of pNPG4 and reduced the release of pNPG2 and pNPG3. In the reaction with amylose EX-1, the mutants other than I246A and Y248A exhibited lower *k*_cat_/*K*_m_ values than the WT (5.0–94% of the WT value). L175A, which exhibited lower activity toward blocked pNPG7 than the WT, showed the lowest *k*_cat_/*K*_m_ among the mutant enzymes. W77A and K210A showed respectively 3.9- and 5.2-fold lower *k*_cat_/*K*_m_ values than the WT, although both mutants showed 2-fold higher activity toward blocked pNPG7 than the WT. Y248A showed a 2.4-fold higher *k*_cat_/*K*_m_ for amylose EX-1 than that of the WT. This mutant showed 6.0- and 2.5-fold lower *K*_m_ and *k*_cat_ than those of the WT, respectively.

#### 2.4.2. Functional Analysis of Maltose-Binding Residues

**Figure 8:**
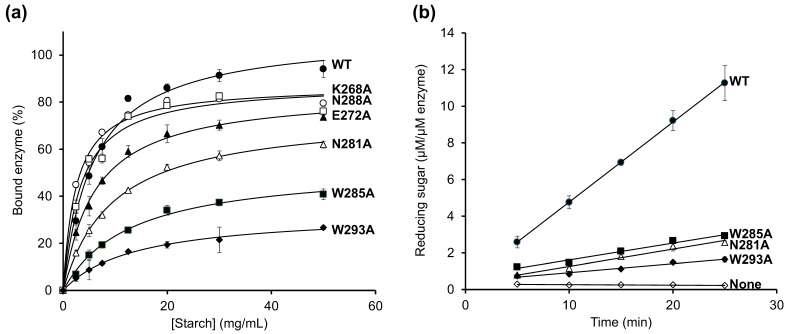
Binding and degradation assay of AMA variants for maize starch granules. (**a**) Binding assay. (**b**) Time course of degradation of 10 mg/mL starch granules. Values and error bars are average and standard deviation for three independent experimental values. Black circles, WT; white circles, K268A; black triangles, E272A; white triangles, N281A; black square, W285A; white square, N288A; black diamonds, W293A; and white diamond, no enzyme.

The residues located near the maltose bound to the β→α loop 6 were substituted with Ala. Among the AMA mutants, N281A, W285A, and W293A were only slightly adsorbed onto the β-CD Sepharose 6B column (the adsorption rates for these enzymes were 0.85–10.1%). In the binding assay of AMA variants to maize starch granules, the bound enzyme ratio followed a saturation curve against the starch granule concentration (Figure 8a). N281A, W285A, and W293A showed 1.65–2.81-fold higher *K*_d_ values than the WT (Table 5). W285A and W293A exhibited significantly lower *B*_max_ values than the WT and the other mutants, K268A, E272A, and N288A, which showed similar or lower *K*_d_ values than the WT.

All mutants of the maltose-binding site had 2.1- to 5.8-fold higher activity toward 2 mM blocked pNPG7 and similar or 1.5- to 2.2-fold higher *k*_cat_/*K*_m_ for amylose EX-1 than the WT (Table 6). However, N281A, W285A, and W293A, which showed higher *K*_d_ values for starch granules than the WT, degraded starch granules at 4.3- to 8.9-fold lower rates than the WT (Figure 8b).

## 3. Discussion

Insect-derived GH13_15 α-amylases are important for pest regulation and the efficient production of beneficial insects such as honeybees and silkworms. Honey α-amylase, which degrades food starch [13], is considered as honeybee α-amylase because it shows different properties from nectar and pollen α-amylases [10] and the peptide fragment of AMA is present in the tryptic digest of honey proteins [14]. In this study, we demonstrated that honey α-amylase is unambiguously AMA via LC–MS/MS analysis of tryptic peptides from the purified honey α-amylase. The recombinant AMA produced in *P. pastoris* exhibited an optimum pH, pH stability, and thermal stability similar to those of honey α-amylase [12], supporting this conclusion.

**Figure 9:**
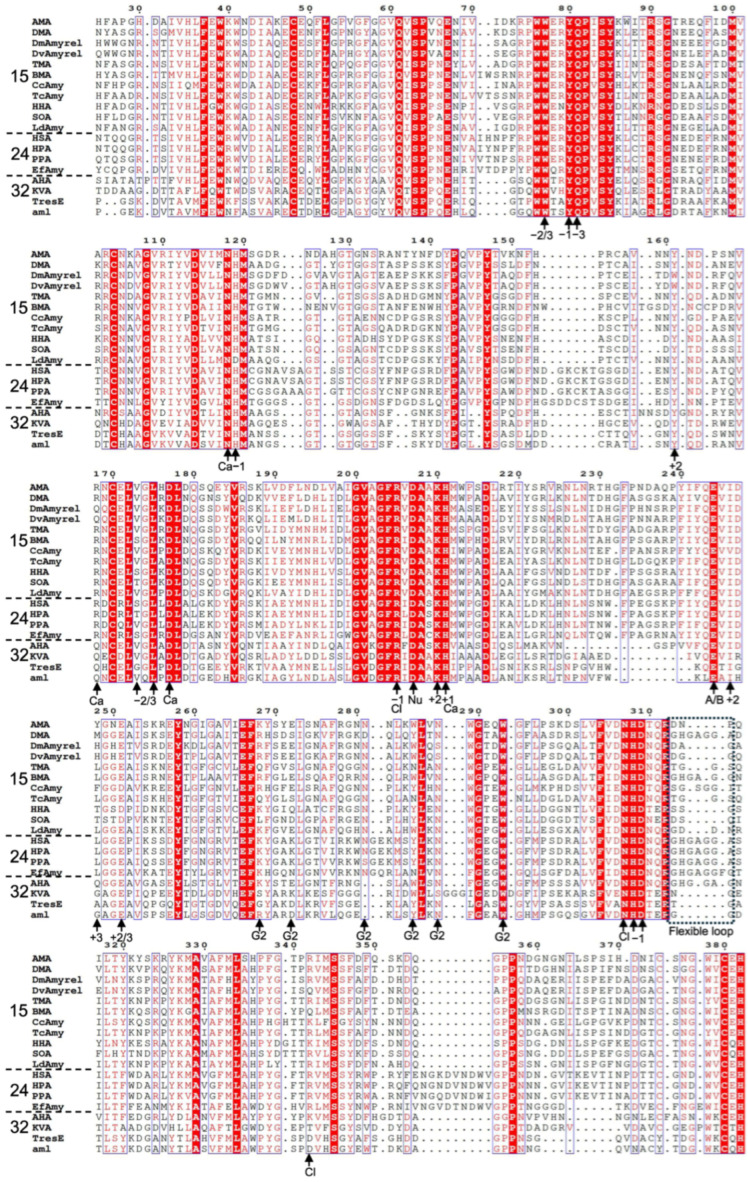
Multiple-sequence alignment of α-amylases of GH13_15, GH13_24, and GH13_32. AMA, *A. mellifera* α-amylase (NP_001011598.1); DMA, *D. melanogaster* α-amylase (AAA92226.1) [19]; DmAmyrel, *D. melanogaster* α-amylase (AAK93479.1) [26]; DvAmyrel, *Drosophila virilis* α-amylase (AAF61427.1) [26]; TMA, *T. molitor* α-amylase (P56634) [28]; BMA, *Bombyx mori* α-amylase (ACT64133.1) [29]; CcAmy, *Callosobruchus chinensis* α-amylase (KU641476) [30]; TcAmy, *Tribolium castaneum* α-amylase (NM_001114376.1) [30]; HHA, *Hypothenemus hampei* α-amylase (AHY03307.1) [31]; SOA, *Sitophilus oryzae* α-amylase (HQ158012.1) [32]; LdAmy, *Leptinotarsa decemlineata* (XP_023015287.2) [18]; HSA, human (*Homo sapiens*) salivary α-amylase (AAH63129.1) [33]; HPA, human pancreatic α-amylase (AAA51724.1) [34]; PPA, porcine (*Sus scrofa*) pancreatic α-amylase (AAF02828.1) [35]; EfAmy, *Eisenia fetida* α-amylase (BAV13234.1) [36]; AHA, *Pseudoalteromonas haloplanktis* α-amylase (CAA41481.1) [37]; KVA*, Kocuria varians* α-amylase (BAJ52728.1) [38]; TresE, *Streptomyces dimorphogenes* α-amylase (BDS38158.1) [39]; aml, *Streptomyces venezuelae* α-amylase (AAB36561.1) [40]. Multiple-sequence alignment was constructed using MAFFT [41] and visualized using ESPript 3.2 [42]. Residues, forming active center, subsites, maltose (G2) binding site, and Ca^2+^/Cl^−^ binding sites, are indicated by arrows. Subfamily numbers are provided at the left of the alignment. The region of the flexible loop is boxed by a dotted line.

Although the addition of NaCl hardly affected the activity of AMA (Figure 3b), chloride ion binding was observed near the active site of AMA, which was consistent with the structures of DMA [19] and TMA [20] (Figure 6c). Such chloride ion binding is also observed in α-amylases belonging to GH13_24 and GH13_32 [21,22,23,24,25]. The chloride ion is coordinated by well-conserved Arg and Asn (Arg205 and Asn307 of AMA, respectively) in GH13 enzymes [3], together with the second Arg (Arg342 in AMA) in β-strand 8 of domain A, which is substituted with other residues in some enzymes (Figure 9). The *D. melanogaster* α-amylase paralog Amyrel (DmAmyrel) possesses a Gln at the position corresponding to the second Arg, and this enzyme has much lower activity and less dependency on chloride ions than DMA [26]. Considering the conservation of the chloride ion binding residues in AMA, the chloride ion is probably tightly bound to the binding site, and further addition of chloride ions is not required for activation, whereas human pancreatic α-amylase (HPA) significantly increases its reaction rate in the presence of chloride ions (the dissociation constant for the chloride ion is 0.5 mM) [27]. In HPA, the roles of the chloride ion are well characterized through kinetic, mutational, and structural analysis: the chloride ion is responsible for stabilizing the general acid/base Glu and the second catalytic Asp in β→α loops 7 at their proper positions, and for shielding the general acid/base from the positive charge of the Arg in β-strand 8 of domain A [27]. The chloride ion in AMA also presumably maintains a proper active site structure, similar to its role in HPA.

In the reaction with pNPG3–5, AMA produced pNPG2–pNPG4. Similarly, DmAmyrel produces DP n-1 maltooligosaccharides from DP n maltooligosaccharides, whereas DMA does not [43]. DMA possesses a flexible Gly-His-Gly-Ala region in β→α loop 7, and His293 in this region forms a hydrogen bond with d-glucosyl residue at subsite −2 [19], while DmAmyrel and AMA lack this region (Figure 9). Feller et al. reported that the lack of this flexible region in DmAmyrel is responsible for its 4-α-glucanotransferase activity [43]. Thus, pNP-maltooligosaccharides of DP n-1 were presumably produced from pNP-maltooligosaccharides of DP n via a 4-α-glucanotransferase reaction and subsequent hydrolysis rather than exo-wise hydrolysis. To exclude the 4-α-glucanotransferase activity, we used blocked pNP-maltooligosaccharides to determine subsite affinity. The 4,6-benzylidene-d-glucosyl residue of blocked pNPG2–pNPG4 exclusively occupied subsite −2 and the affinities of subsites +1 and +2 were readily determined (Figure 4b). Trp77, located at the intermediate position between subsites −2 and −3 (Figure 6f), is predicted to interact favorably with the 4,6-benzylidene group of blocked pNPG-maltooligosaccharide at subsite −2. Blocked pNP-maltooligosaccharides are not suitable substrates for determining affinity for minus subsites. Therefore, the affinity for minus subsites was determined from BCF and the *k*_cat_/*K*_m_ values for unmodified pNP-maltooligosaccharides. The subsite affinity map of an insect α-amylase was previously investigated using the enzyme from Colorado potato beetles (*Leptinotarsa decemlineata*; LdAmy) [18]. Compared with the subsite affinity map of LdAmy, AMA showed a similar affinity for subsites −3 and +2, although it exhibited a higher affinity for subsite −2. These subsite affinity maps are also similar to those of GH13_24 mammalian α-amylases, such as human salivary α-amylase (HSA) and porcine (*Sus scrofa*) pancreatic α-amylase (PPA) (Figure 5). Consistent with this, the substrate-binding residues of AMA were well conserved in GH13_15 and GH13_24, except for Tyr248 in AMA (Figure 9). GH13_32 enzymes, including the chloride ion-dependent α-amylase from *Pseudoalteromonas haloplanktis* [25] and the mammalian α-amylase-specific proteinaceous inhibitor tendamistat-sensitive α-amylase from *Streptomyces venezuelae* [40], also conserve these substrate-binding residues (Figure 9), indicating that GH13_32 enzymes share the same substrate-binding mechanism. In this study, we performed Ala substitution of the residues at subsites −3, −2, +2, and +3 of AMA to characterize their roles, and demonstrated that Gln81 and Leu175, which are conserved across the three subfamilies of GH13 (Figure 9), are important for activity toward both blocked pNPG7 and amylose EX-1 (Table 4). Notably, the L175A mutation resulted in a significant reduction in *k*_cat_/*K*_m_ for amylose EX-1. Ala substitutions of the other residues increased the activity toward blocked pNPG7 by 1.9- to 9.3-fold. The Y161A mutation enhanced this activity most significantly, although the Tyr/Trp aromatic residue was conserved at this position. In contrast to the activity toward blocked pNPG7, the W77A and K210A mutations severely decreased *k*_cat_/*K*_m_ for amylose EX-1 (Table 4), suggesting that these residues are important for the proper binding of long-chain substrates. These residues are conserved across the three subfamilies, suggesting their similar functions. The Y248A mutation enhanced *k*_cat_/*K*_m_ for amylose EX-1 by 2.4-fold. Because Y248A showed a 6.0-fold lower *K*_m_ for amylose EX-1, Tyr248 likely destabilized the Michaelis complex during reactions with amylose EX-1. A residue smaller than Tyr is frequently found at this position in other enzymes (Figure 9), and such a smaller residue may stabilize the transition state by stabilizing the Michaelis complex. To understand the mechanism of the activity enhancement observed in the Ala-substituted mutants, further mutational and structural analyses are required. Amino acid sequences of putative Hymenoptera α-amylases are compared (Figure S2). Among five putative α-amylases, three enzymes possess the flexible loop sequence corresponding to that of DMA, suggesting that the lack of the flexible loop observed in AMA is not conserved in Hymenoptera α-amylases. Additionally, a substitution of residues corresponding to Trp77, Tyr161, Val173, and Tyr248, of AMA was observed in the Hymenoptera α-amylases. A functional analysis of these putative is expected to provide fruitful information for understanding insect α-amylases.

Our structural analysis of AMA indicates the presence of another sugar-binding site formed by β→α loop 6 (Figure 6g). Ala substitution of the residues forming this sugar-binding site increased the activity toward blocked pNPG7 and amylose EX-1 (Table 6), although the reason for this activity increase remains unclear. This sugar-binding site does not play a direct role in the reactions with these substrates. However, Ala substitutions of Asn281, Trp285, and Trp293 at this site clearly reduced the binding affinity and degradation activity toward starch granules (Table 5 and Table 6 and Figure 8), indicating that this sugar-binding site serves as a binding site for starch granules. Binding to starch granules is required before degradation. As N281A, W285A, and W293A mutants hardly adsorbed onto β-CD Sepharose, the affinity of AMA toward this affinity resin is mediated by the sugar-binding site in β→α loop 6. This sugar-binding site of β→α loop 6 is shared by PPA and HPA [44,45]. In HPA, this site, termed the surface-binding site 7, contains a WY clamp consisting of Tyr276 and Trp284, which correspond to Trp285 and Trp293 of AMA, respectively [45]. Similar to the results for AMA, the Y276A and W284A mutations in HPA hardly affected the catalytic efficiency for oligosaccharides and soluble starch, but decreased the binding affinity for starch granules. Sequence comparison of the GH13_15, GH13_24, and GH13_32 enzymes shows that only Trp293 of AMA is conserved among the three residues responsible for binding to starch granules (Figure 9). Notably, GH13_32 enzymes lack a residue corresponding to Asn281 in AMA. In *P. haloplanktis* α-amylase, Gly236 is located at a position similar to Asn281 of AMA [25]. α-Amylase from *Sitophilus oryzae* (SOA) is reported to degrade starch granules [32], and this enzyme contains Asn275, Tyr279, and Trp287 at the positions corresponding to Asn281, Trp285, and Trp293 in AMA, respectively. SOA Tyr279 is expected to complement the function of AMA Trp285 because HPA contains Tyr276 as a sugar-binding residue at this position [45]. We propose that the Asn-Trp/Tyr-Trp triad (positions 281, 285, and 293 in AMA) is a functional set involved in the degradation of starch granules. This functional set is useful for predicting the starch granule degradation ability of insect α-amylases.

## 4. Materials and Methods

### 4.1. Substrates

#### 4.1.1. Commercial Substrates

The following substrates were purchased: soluble starch (amylase assay grade), Nacalai Tesque (Kyoto, Japan); amylose EX-1, Nagase Viita (Okayama, Japan); blocked pNPG7 and pNPG6, Sigma-Aldrich (Burlington, MA, USA); pNP α-d-glucopyranoside (pNPG) for the enzyme assay, Fujifilm Wako Pure Chemical (Osaka, Japan). pNPG5 and pNPG7 were gifted by the Research Institute of Nihon Shokuhin Kako (Tokyo, Japan).

#### 4.1.2. Preparation of pNPG

pNP 2,3,4,6-tetra-*O*-acetyl α-d-glucoside was synthesized as follows: 1,2,3,4,6-penta-*O*-acetyl-β-d-glucopyranose (117 g, 300 mmol; Tokyo Chemical Industry, Tokyo, Japan) and pNP (46 g, 330 mmol; Fujifilm Wako Pure Chemical) were dissolved in 110 mL of dichloromethane. SnCl_4_ (81 g; Sigma-Aldrich) was gradually added to the mixture and refluxed at 60 °C for 3 h. The reaction was terminated by adding crushed ice with NaHCO_3_, and the sample was filtered through Celite No. 535 (Fujifilm Wako Pure Chemical). The filtrate was washed with saturated NaCl and the dichloromethane layer was dried over MgSO_4_ and evaporated in vacuo. pNP 2,3,4,6-tetra-*O*-acetyl α-d-glucopyranoside was purified using silica gel column chromatography using hexane/ethyl acetate (1:1, *v*/*v*) as the mobile phase. Fractions containing pNP 2,3,4,6-tetra-*O*-acetyl α-d-glucoside were evaporated in vacuo, and crystalline pNP 2,3,4,6-tetra-*O*-acetyl α-d-glucoside (35.8 g, 76.3 mmol, 25% yield) was obtained by adding hexane. All crystalline products were suspended in 350 mL of methanol, and sodium methoxide was added until the pH reached 9. The reaction was performed at room temperature (22–25 °C) with stirring until deacetylation was complete. The sample was deionized using AG 50W-X8 resin (Bio-Rad, Hercules, CA, USA) and evaporated in vacuo. Crystalline pNPG (19.3 g, 64 mmol, 84% yield) was obtained by sample evaporation.

#### 4.1.3. Preparation of pNP-Maltooligosaccharides

A reaction mixture (600 mL) consisting of 33 mM pNPG, 200 g/L soluble starch, 20 mM sodium acetate (pH 6.0), and 1 μL/mL cyclodextrin glucanotransferase (Contizyme; Amano Enzyme, Nagoya, Japan), was incubated at 50 °C for 24 h. The produced pNP-maltooligosaccharides were adsorbed onto an FPX66 column (2.6 cm I.D. × 60 cm; Organo, Tokyo, Japan) and eluted sequentially with water (1.8 L) and 20% (*v*/*v*), 30% (*v*/*v*), and 40% (*v*/*v*) ethanol (1 L each). The collected fractions were evaporated in vacuo and separated using gel filtration column chromatography with a Toyopearl HW40S column (5 cm I.D. × 110 cm; Tosoh, Tokyo, Japan) using water as the mobile phase. The purified pNPG2–5 (459–1740 mg) was lyophilized.

#### 4.1.4. Preparation of Blocked pNP-Maltooligosaccharides

Blocked pNP-maltooligosaccharides were synthesized as described previously [46]. The precursor pNP-maltooligosaccharides (pNPG2, 0.464 mmol; pNPG3 and pNPG4, 0.232 mmol; and pNPG5–7, 0.116 mmol) were dissolved in *N*,*N*-dimethylformamide to a final concentration of 72.5 mM. Five equivalents of benzaldehyde dimethyl acetal and 0.74 equivalents of *p*-toluensulfonic acid monohydrate were added, and the mixture was incubated with stirring in vacuo (100 Pa) at 50 °C for 3 h. An equal amount of benzaldehyde dimethyl acetal was added, and the mixture was further incubated with stirring at 50 °C for 3 h. The resulting blocked pNP-maltooligosaccharides were purified on a Duolite S876 column (1.6 cm I.D. × 13 cm; Sumitomo Chemical, Tokyo, Japan) and equilibrated with water. The adsorbed blocked pNP-maltooligosaccharides were eluted by increasing ethanol concentration. The fractions containing blocked pNPG2 and pNPG3 were evaporated and dissolved in methanol, whereas the other blocked pNP-maltooligosaccharides were lyophilized. The yield of blocked pNP-maltooligosaccharides ranged from 30 to 69%.

### 4.2. Identification of Honey α-Amylase

Commercial honey (50 g) was diluted with 50 mL of 10 mM sodium acetate (pH 6.0) and dialyzed against the same buffer. The resulting samples were separated using anion-exchange column chromatography with a DEAE Sepharose Fast Flow column (Cytiva, Tokyo, Japan; 2.6 cm I.D. × 5 cm) equilibrated with 20 mM sodium phosphate (pH 7.0). After the sample application, the non-adsorbed protein fraction was eluted with equilibration buffer and collected. Ammonium sulfate was added to the collected sample up to 30% saturation, and the sample was separated by hydrophobic interaction column chromatography using a Toyopearl Butyl-650M column (Tosoh; 2.6 cm I.D. × 4 cm) equilibrated with 10 mM sodium phosphate (pH 7.0) containing 30% saturation ammonium sulfate. After washing the non-adsorbed proteins with equilibration buffer, the adsorbed proteins were eluted using a linear gradient of 30–0% saturation ammonium sulfate (total elution volume, 200 mL). The collected fractions were concentrated to 0.0953 mg/mL using a 10 kDa cutoff centrifugal device (Vivaspin YM-30 concentrator; Sartorius, Göttingen, Germany).

The purified protein (1 μg) was separated via SDS-PAGE. The AMA band was excised, and the protein was digested with trypsin using an In-Gel Tryptic Digestion Kit (Thermo Fisher Scientific, Waltham, MA, USA). The resulting peptides were purified using a Pierce C18 Spin Column (Thermo Fisher Scientific) and dissolved in 15 μL of 0.1% (*v*/*v*) formic acid. An aliquot (4 μL) was analyzed via LC–MS/MS using an EASY-nLC1000 system (Thermo Fisher Scientific) equipped with an NTCC-360/75-3-125 C18 column (0.075 mm I.D. × 120 mm; Nikkyo Technos, Tokyo, Japan). Elution was performed using a gradient of 0–90% (*v*/*v*) acetonitrile in 0.1% (*v*/*v*) formic acid at a flow rate of 300 nL/min under the following conditions: 0 min, 0%; 1 min, 5%; 21 min, 35%; 23 min, 90%; and 40 min, 90%. Mass spectrometry was performed using an Orbitrap Elite Hybrid Ion Trap-Orbitrap Mass Spectrometer (Thermo Fisher Scientific). The data were processed using the Proteome Discoverer software (Thermo Fisher Scientific).

### 4.3. Production and Purification of Recombinant AMA

#### 4.3.1. Expression of AMA in *E. coli*

The synthesized AMA gene, codon-optimized for *E. coli* expression (Genscript, Piscataway, NJ, USA; Figure S3), was cloned into pET-23a (Novagen, Darmstadt, Germany). Met and Leu-Glu-His-His-His-His-His-His were attached to the N- and C-termini, respectively, of the mature AMA (Glu18–Ala493). The AMA gene was amplified using a primer set (5′-AAGAAGGAGATATACATATGGAAATAGCACATAATGATCC-3′ and 5′-CAGTGGTGGTGGTGGTGGTGCTCGAGCGCCATTTTCGCCTTAACAT-3′) and Primestar Max DNA polymerase (Takara Bio, Kusatsu, Japan). The fragment was assembled with pET-23a amplified with primers 5′-TTGCGGCCGCACTCGAGCACC-3′ and 5′-GCCATATGTATATCTCCTTCT-3′ using NEBuilder HiFi DNA Assembly Master Mix (New England Biolabs, Ipswich, MA, USA). The resulting plasmid sequence was verified by sequencing (Eurofin Genomics, Tokyo, Japan). An *E. coli* SoluBL21 transformant (Amsbio, Oxfordshire, UK) carrying the expression plasmid was cultivated in 20 mL of LB broth containing 100 μg/mL ampicillin until A600 reached 0.5. Recombinant protein expression was induced by adding isopropyl β-thiogalactopyranoside to a final concentration of 0.1 or 0.5 mM, and induction was carried out at 18 °C or 30 °C for 24 h. Bacterial cells were harvested from 1 mL of culture broth using centrifugation (15,000× *g*, 4 °C, 1 min) and suspended in water (A600 was adjusted to 10). This suspension and the cell-free extract prepared via sonication (10 μL each) were analyzed using SDS-PAGE to evaluate the expression level of recombinant AMA.

#### 4.3.2. Production and Purification of AMA WT in *P. pastoris*

A synthesized AMA gene optimized for *P. pastoris* expression (Genscript) was cloned into pPICZαA (Invitrogen, Carlsbad, CA, USA; Figure S3) between the α-factor signal sequence and the c-myc epitope sequence. The DNA fragment of the AMA gene was amplified using a primer set (5′-AGAGAGGCTGAAGCTGAGATTGCACATAATGATCC-3′ and 5′-GATGAGTTTTTGTTCTCAAGCCATCTTAGCCTTGACAT-3′) and Primestar HS DNA polymerase (Takara Bio) and assembled with a pPICZαA fragment, amplified with primers 5′- GAACAAAAACTCATCTCAGAAGAGGATCT-3′ and 5′- AGCTTCAGCCTCTCTTTTCTCGAGAGATA-3′. The sequence was verified as described above. The expression plasmid (10 μg) was linearized via SacI digestion and introduced into *P. pastoris* X-33 cells by electroporation using a Gene Pulser (Bio-Rad).

Transformants selected on a YPDSZ plate (10 mg/mL yeast extract, 20 mg/mL peptone, 20 mg/mL d-glucose, 1 M d-glucitol, 20 mg/mL agar, and 100 μg/mL zeocin) were cultivated in 800 mL of BMGY medium (10 mg/mL yeast extract, 20 mg/mL peptone, 13.4 mg/mL yeast nitrogen base, 4 μg/mL biotin, 10 mg/mL glycerol, and 0.1 M potassium phosphate buffer, pH 6.0) at 30 °C for 18 h. Yeast cells harvested using centrifugation (2300× *g*, room temperature, 5 min) were resuspended in 800 mL of BMMY medium (where glycerol in BMGY was replaced with 0.5% *v*/*v* methanol) and incubated at 30 °C with vigorous shaking for 96 h to induce recombinant enzyme expression. Methanol was added at a final concentration of 0.5% (*v*/*v*) every 24 h to maintain the induction. The culture supernatant was obtained using centrifugation (6000× *g*, 4 °C, 3 min) and applied to a β-CD Sepharose 6B column (1.6 cm I.D. × 7 cm) prepared as described previously [47]. The column was equilibrated with 10 mM sodium acetate (pH 6.0) containing 5 mM CaCl_2_ and 50 g/L ammonium sulfate. After washing the column with the equilibration buffer, the adsorbed protein was eluted with the equilibrium buffer containing 10 g/L β-CD (Fujifilm Wako Pure Chemical). The collected fractions were concentrated to 10 mL as described above and further purified using gel filtration column chromatography with a Toyopearl HW-55S column (Tosoh; 2.6 cm i.d. × 100 cm) equilibrated with 10 mM HEPES-NaOH (pH 7.0). The concentration of recombinant AMA was determined by amino acid analysis using an L-8900 amino acid analyzer (Hitachi High-Technologies, Tokyo, Japan) after complete hydrolysis of the purified protein in 6 M HCl at 110 °C for 24 h.

#### 4.3.3. Preparation of AMA Mutants

Expression plasmids for AMA mutants were prepared using a PrimeStar Mutagenesis Basal Kit (Takara Bio) with primers listed in Table S1 and the WT plasmid as a template. Plasmid DNA sequences were confirmed by sequencing. Mutant enzymes were produced in *P. pastoris* and purified in the same manner as the WT, but the purified samples were dialyzed against 10 mM HEPES-NaOH (pH 7.0) containing 5 mM CaCl_2_ instead of gel filtration column chromatography. Because the N281A, W285A, and W293A mutants did not adsorb onto the β-CD Sepharose 6B column, they were purified using Toyopearl Butyl-650M and DEAE Sepharose Fast Flow column chromatography as described in Section 4.2. The concentrations of the mutant enzymes were determined using the specific coefficient of WT at A280 (1.93 for 1 mg/mL and 0.105 for 1 μM).

### 4.4. Standard Enzyme Assay

Activity toward 2 mM blocked pNPG7 was measured using an α-amylase assay kit (Ceralpha method; Megazyme, Bray, Ireland). Enzyme samples were diluted with 10 mM sodium acetate (pH 5.0) containing 1 g/L bovine serum albumin (BSA; Nacalai Tesque). The enzymatic reaction was performed at 37 °C for 10 min and terminated by adding a two-fold volume of 1 M Na_2_CO_3_ relative to the reaction mixture. The concentration of liberated *p*-nitrophenol (pNP) was quantified using a molar extinction coefficient of 16,700. One unit (U) of enzyme activity was defined as the amount of enzyme that produces 1 μmol of pNP per 1 min under these conditions.

### 4.5. Effects of pH and Temperature on Activity and Stability

The optimum pH was determined based on the reaction rates at various pH values. A reaction mixture consisting of 4 g/L soluble starch, 40 mM buffer (sodium acetate for pH 3.3–5.9, MES-NaOH for pH 5.9–6.9, and HEPES-NaOH for pH 6.9–7.8), 2 mM CaCl_2_, 0.2 g/L BSA, and 10.6 nM AMA was incubated at 37 °C for 10 min. The reducing sugars produced were quantified using the dinitrosalicylic acid method [48] with d-glucose as the standard. The pH stability was determined based on the residual activity after incubating 212 nM AMA in 0.1 M buffer (sodium acetate for pH 3.0–5.4, MES-NaOH for pH 6.3, HEPES-NaOH for pH 7.3 and 8.1, and Glycine-NaOH for pH 9.2–11.0) at 4 °C for 24 h. The thermal stability range was determined from the residual activity after incubating 26.5 nM AMA in 10 mM sodium acetate (pH 5.0) containing 1 g/L BSA at 30–60 °C for 15 min. The residual activity was measured using a standard enzyme assay. The stable range was defined as the range in which AMA retained ≥97% of its original activity. All experiments were conducted in triplicates.

### 4.6. Effects of Calcium and Chloride Ions on Activity and Stability

Reaction rates for 4 g/L soluble starch in 42 mM sodium acetate (pH 5.0) containing 0–2 mM CaCl_2_ or 0–20 mM NaCl were measured as described in Section 4.5. AMA (1.06 μM) in 10 mM HEPES-NaOH (pH 7.0) was subjected to DSC using a MicroCal PEAQ-DSC Automated Microcalorimeter (Malvern Panalytical, Malvern, Worcestershire, UK) over a temperature range of 20–80 °C at a scan rate of 60 °C/h. Samples without AMA were used as references, and the data were fitted to a non-two-state model using PEAQ-DSC software (Malvern Panalytical). Samples containing 2 mM CaCl_2_ or 2 mM EDTA-2Na were analyzed in triplicates using the same method.

### 4.7. Kinetic Parameters

#### 4.7.1. Amylose EX-1

A reaction mixture containing the enzyme, 42 mM sodium acetate (pH 5.0), 0.2–2.8 mg/mL amylose EX-1, 2 mM CaCl_2_, and 0.02 mg/mL BSA was incubated at 37 °C. Aliquots of the reaction mixture were collected every 4 min, and the reducing sugars were quantified using the bicinchoninic acid method [49] with maltose as a standard. Reaction rates were calculated from the resulting progress curves, and the kinetic parameters of the Michaelis–Menten equation were determined using nonlinear regression analysis using Grafit version 7.0.2 (Erithacus Software, East Grinstead, UK).

#### 4.7.2. pNP-Maltooligosaccharides

A reaction mixture, containing the enzyme, 42 mM sodium acetate (pH 5.0), 0.00781–1 mM substrate (pNPG, blocked or non-modified pNPG2–pNPG7), 2 mM CaCl_2_, 0.02 mg/mL BSA was incubated at 37 °C for 10 min (260 min for pNPG). The reaction was terminated by adding half the volume of 2 M Na_2_CO_3_. For pNPG, pNPG2, blocked pNPG2, and pNPG3 (pNP release), the liberated pNP was measured as described in Section 4.4. For pNPG3–pNPG7, the reaction products were quantified using high-performance liquid chromatography (HPLC) under the following conditions: column, Unison UK-C18 (4.6 mm I.D. × 250 mm; Imtakt, Kyoto, Japan); column temperature, 50 °C; eluent, 9% acetonitrile; flow rate, 1 mL/min; detection, A313; injection volume, 10 μL. For blocked pNPG3–pNPG7, the HPLC conditions were as follows: column, Capcell Pac C18 AQ (4.6 mm I.D. × 250 mm; Osaka Soda, Osaka, Japan); column temperature, 50 °C; elution, 5–20% acetonitrile (20 min), 20–90% acetonitrile (20–25 min); flow rate, 1 mL/min; detection, A313; injection volume, 10 μL. Kinetic parameters for the Michaelis–Menten equation were determined from sum of reaction velocities for respective reaction products. The BCF was calculated as the ratio of the production rate of each product to the total reaction rate. The kinetic parameters were determined via nonlinear regression, as described above.

### 4.8. Binding and Degradation Assay for Starch Granules

The binding affinity to starch granules was evaluated based on the amount of enzyme adsorbed onto the starch granules according to the following equation [45,50]:

[E]_bound_/[E]_0_ (%) = *B*_max_[Starch]/([Starch] + *K*_d_)

AMA derivatives (0.25 μM) were incubated in 50 mM sodium acetate (pH 5.0) containing 0–50 mg/mL starch granules from maize (Sigma-Aldrich) on ice for 30 min with occasional vortexing. The amount of enzyme in the supernatant, obtained using centrifugation (15,000× *g*, 4 °C, 5 min), was determined as non-bound enzyme based on hydrolytic activity to soluble starch, which was measured as described Section 4.5.

The degradation velocity of the starch granules was determined from the progress curves for reducing sugar production. A reaction mixture (1 mL) consisting of 0.206–0.602 μM enzyme, 42 mM sodium acetate (pH 5.0), 2 mM CaCl_2_, 0.02 mg/mL BSA, and 10 mg/mL starch granules was incubated at 37 °C with voltexing every min. Aliquots (0.1 mL) were collected every 5 min and the reducing sugars produced were measured as described in Section 4.5.

### 4.9. Crystallization and Data Collection

Purified AMA was concentrated to 10 mg/mL using a 10 kDa cutoff centrifugal device (Vivaspin YM-10 concentrator, Sartorius). Crystallization of AMA was achieved at 20 °C within 20 days via sitting drop vapor diffusion. Protein drops were prepared by mixing equal volumes (0.75 μL each) of the protein solution and crystallization buffer. AMA crystals were obtained from a reservoir solution consisting of 0.1 M imidazole-HCl (pH 8.0), 40% (*w*/*v*) polyethylene glycol, and 10 mM acarbose (Carbosynth, Compton, Berkshire, UK).

For X-ray diffraction, crystals were picked from the crystallization solution and flash-cooled in liquid nitrogen. Diffraction data were automatically collected by the ZOO system [51,52] at beamline BL-45XU at SPring-8 (Hyogo, Japan). The data were processed (indexing, integration, scaling, and merging) using the XDS package [53]. The asymmetric unit contained a single molecule. The Matthews coefficient [54] was 2.41 Å^3^/Da, which corresponded to an estimated solvent content of 49.0%. The data collection statistics are summarized in Table 3.

### 4.10. Structural Determination and Refinement

The structure was determined by molecular replacement using Phaser-MR in the Phenix suite [55], employing the AMA model generated using ColabFold [56] as the search model. Several rounds of refinement were performed using Phenix.refine in the Phenix Program Suite. The protein structure model was iteratively adjusted by manual fitting and rebuilding based on 2*F*_o_ − *F*_c_ and *F*_o_ − *F*_c_ electron density maps using COOT [57]. Water molecules and ligands were modeled according to 2*F*_o_ − *F*_c_ and *F*_o_ − *F*_c_ electron densities. The final refinement statistics and geometric parameters, evaluated using MolProbity [58], are presented in Table 3. Structural illustrations were generated using PyMol ver. 2.2.0 (Schrödinger, LLC, New York, NY, USA).

## Figures and Tables

**Table 1:** Thermodynamic parameters of AMA denaturation.

Additive	Δ*H*_cal_ (kJ/mol)	Δ*H*_VH_ (kJ/mol)	Δ*H*_cal_/Δ*H*_VH_	*T*_m_ (°C)
None	1050 ± 40	379 ± 20	2.77	55.6 ± 0.5
CaCl_2_	1390 ± 70	536 ± 12	2.59	68.3 ± 0.1
NaCl	972 ± 111	323 ± 12	3.01	55.9 ± 0.1

Values are average ± standard deviation for three independent experimental values.

**Table 2:** Kinetic parameters of AMA.

Substrate	*k*_cat_ (s^−1^)	*K*_m_ (mM)	*k*_cat_/*K*_m_ (s^−1^ mM^−1^)
pNP α-d-glucopyranoside	N.D.	N.D.	1.06 × 10^−4^
pNPG2	N.D.	N.D.	0.108
pNPG3 (pNP release)	8.98 ± 0.57	2.25 ± 0.28	4.00
pNPG6	327 ± 17	0.0393 ± 0.0039	8310
pNPG7	333 ± 39	0.0369 ± 0.0095	9030
Blocked pNPG2	N.D.	N.D.	0.317
Blocked pNPG3	10.1 ± 0.2	1.05 ± 0.02	9.67
Blocked pNPG4	131 ± 3	0.0943 ± 0.008	1390
Blocked pNPG5	83.1 ± 1.3	0.0256 ± 0.0021	3250
Blocked pNPG6	111 ± 3	0.00576 ± 0.00144	19,300
Blocked pNPG7	104 ± 6	0.00708 ± 0.00130	14,600
Amylose EX-1 (DP17)	395 ± 13	0.461 ± 0.06 ^1^	857 ^2^

^1^ mg mL^−1^. ^2^ s^−1^mg^−1^mL. Data are average ± standard deviation for three independent experiments.

**Table 3:** Data collection and refinement statistics.

		AMA
PDB entry		26WD
Data collection	Beamline	SPring-8 BL45XU
	Space group	*P*4_1_ 2_1_ 2
	Unit cell parameters *a*, *b*, *c*, (Å)	89.70, 89.70, 122.19
	Wavelength (Å)	1.00
	Resolution range (Å)	50–1.90 (2.01–1.90)
	Total number of reflections	2,113,534 (342,786)
	Number of unique reflections	40,052 (6355)
	*R*_meas_ (%) ^1^	54.7 (165)
	<*I*/σ(I)>	13.7 (3.80)
	CC_1/2_ (%)	99.7 (58.6)
	Completeness (%)	99.8 (99.9)
	Redundancy	52.8 (53.9)
Refinement	Number of reflections used	39,944
	Number of reflections for *R*_free_	1998
	*R*_work_/*R*_free_ (%) ^2^	20.3/22.9
	Number of atoms	
	Macromolecules	3812
	Ligand	198
	Water	146
	Macromolecules	20.4
	Ligand	29.8
	Water	25.1
	RMSD from ideal	
	Bond lengths (Å)	0.003
	Bond angles (°)	0.62
	Ramachandran	
	Favored (%)	97.25
	Allowed (%)	2.75
	Outliers (%)	0

Values in parentheses are for the highest resolution shell. ^1^ R_meas_ = Σ_hkl_ {*N*(*hkl*)/[*N*(*hkl*) − 1]}^1/2^ Σ_i_ | *I*_i_(*hkl*) − <*I*(*hkl*)> |/Σ*_hkl_* Σ*_i_ I_i_*(*hkl*), where <*I*(*hkl*)> and *N*(*hkl*) are the mean intensity of a set of equivalent reflections and the multiplicity, respectively. ^2^ *R*_work_ = Σ*_hkl_* ||*F_obs_*| − |*F_calc_*||/Σ*_hkl_* |*F*_obs_|, *R*_free_ was calculated for 5% randomly selected test sets that were not used in the refinement.

**Table 4:** Kinetic parameters for AMA mutants of subsites −3, −2, +2, and +3.

Mutant	Subsite Mutated	Blocked pNPG7 ^1^	Amylose EX-1		
		(s^−1^)	*k*_cat_ (s^−1^)	*K*_m_ (mg/mL)	*k*_cat_/*K*_m_ (s^−1^ mg^−1^ mL)
WT	N.A.	87.7 ± 0.8	395 ± 13	0.461 ± 0.064 ^1^	857
W77A	−3/−2	189 ± 8	163 ± 5	0.750 ± 0.067 ^1^	217
Q81A	−3/−2	51.9 ± 0.3	132 ± 13	0.228 ± 0.018 ^1^	579
V173A	−3/−2	171 ± 16	198 ± 11	0.277 ± 0.033 ^1^	715
L175A	−3/−2	34.5 ± 1.0	15.9 ± 0.4	0.369 ± 0.003 ^1^	43.1
Y161A	+2	817 ± 12	420 ± 18	0.579 ± 0.025 ^1^	725
K210A	+2	185 ± 1	134 ± 8	0.813 ± 0.063 ^1^	165
I246A	+2	301 ± 14	290 ± 8	0.285 ± 0.009 ^1^	1020
E251A	+2/+3	356 ± 3	301 ± 17	0.374 ± 0.056 ^1^	805
Y248A	+3	184 ± 3	161 ± 4	0.0774 ± 0.0140 ^1^	2080

^1^ Velocity at 2 mM. Values of WT are from Table 2. Data are average ± standard deviation for three independent experiments. N.A., not applicable.

**Table 5:** Binding parameters of AMA mutants of the maltose-binding site to starch granules.

Enzyme	*B*_max_ (%)	*K*_d_ (mg/mL)
WT	109 ± 3.6	5.81 ± 0.75
K268A	90.8 ± 3.6	2.89 ± 0.53
E272A	84.4 ± 3.6	6.10 ± 0.84
N281A	75.2 ± 2.0	9.58 ± 0.91
W285A	57.6 ± 2.4	15.1 ± 0.4
N288A	94.0 ± 1.2	4.03 ± 0.39
W293A	35.9 ± 1.6	16.3 ± 1.2

Data are average ± standard deviation for three independent experiments.

**Table 6:** Kinetic parameters for AMA mutants of the maltose-binding site.

Enzyme	Blocked pNPG7 ^1^	Amylose EX-1			Starch Granules
	(s^−1^)	*k*_cat_ (s^−1^)	*K*_m_ (mg mL^−1^)	*k*_cat_/*K*_m_ (s^−1^ mg^−1^ mL)	(min^−1^)
WT	87.7 ± 0.8	395 ± 13	0.461 ± 0.064 ^1^	857	0.436 ± 0.052
K268A	185 ± 6	421 ± 23	0.503 ± 0.066 ^1^	837	N. D.
E272A	205 ± 5	616 ± 20	0.443 ± 0.033 ^1^	1390	N. D.
N281A	304 ± 13	367 ± 12	0.194 ± 0.029 ^1^	1890	0.102 ± 0.017
W285A	505 ± 62	350 ± 30	0.275 ± 0.069 ^1^	1270	0.0943 ± 0.0072
N288A	208 ± 22	380 ± 8	0.238 ± 0.011 ^1^	1600	N. D.
W293A	340 ± 7	394 ± 13	0.263 ± 0.041 ^1^	1500	0.0489 ± 0.0066

^1^ Velocity at 2 mM. Values of WT are from Table 2. Data are average ± standard deviation for three independent experiments. N. D., not determined.

## Data Availability

Atomic coordinates and structural factors were deposited in the Protein Data Bank (http://wwpdb.org/; code 26WD).

## Supplementary Materials

The following supporting information can be downloaded at: https://www.mdpi.com/article/10.3390/molecules31152615/s1, Figure S1: HPLC profiles of reaction products of AMA from unmodified and blocked pNP-maltooligosaccharides; Figure S2: Multiple sequence alignment of Hymenoptera putative α-amylases; Figure S3: Synthesized sequence of the AMA gene; Table S1: Sequences of primers for site-directed mutagenesis.

## Abbreviations

The following abbreviations are used in this manuscript:

AMA	Honeybee α-amylase
BCF	Bond cleavage frequency
Blocked pNPGn	*p*-Nitrophenyl 4,6-benzylidene-maltooligosaccharide of DP n
BSA	Bovine serum albumin
CD	Cyclodextrin
DMA	*Drosophila melanogaster* α-amylase
DmAmyrel	*D. melanogaster* α-amylase Amyrel
DP	Degree of polymerization
DSC	Differential scanning calorimetry
GH	Glycoside hydrolase
GHxx	Glycoside hydrolase family xx
GH13_yy	Glycoside hydrolase family 13 subfamily yy
HPA	Human pancreatic α-amylase
HPLC	High-performance liquid chromatography
HSA	Human salivary α-amylase
LC–MS/MS	Liquid chromatography–tandem mass spectrometry
LdAmy	Colorado potato beetle α-amylase
pNP	*p*-Nitrophenol
pNPGn	pNP-maltooligosaccharide of DP n
pNP-maltooligosaccharide	*p*-nitrophenylα-maltooligosaccharide
PPA	Porcine pancreatic α-amylase
TMA	*Tenebrio molitor* α-amylas
