# Supplementary material for: Structure and Function of Honeybee α-Amylase of Glycoside Hydrolase Family 13 Subfamily 15
Source: Molecules. 2026 Jul 27;31(15):2615. doi: 10.3390/molecules31152615 (PMC13468693; doi:10.3390/molecules31152615)
Supplement: Supplementary file 1 [file molecules-31-02615-s001.zip › molecules-4438830-supplementary.pdf]

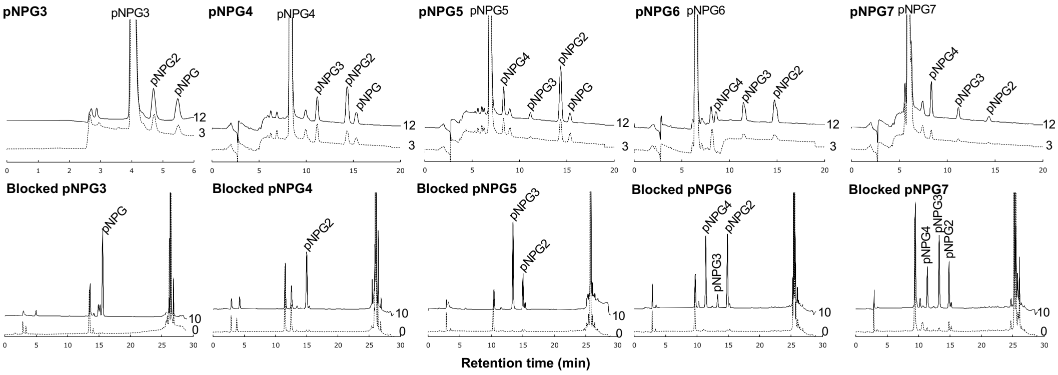

Figure S1. HPLC profiles of reaction products of AMA from unmodified and blocked pNP-maltooligosaccharides. Reaction time (min) is shown in the right of the chromatograms.

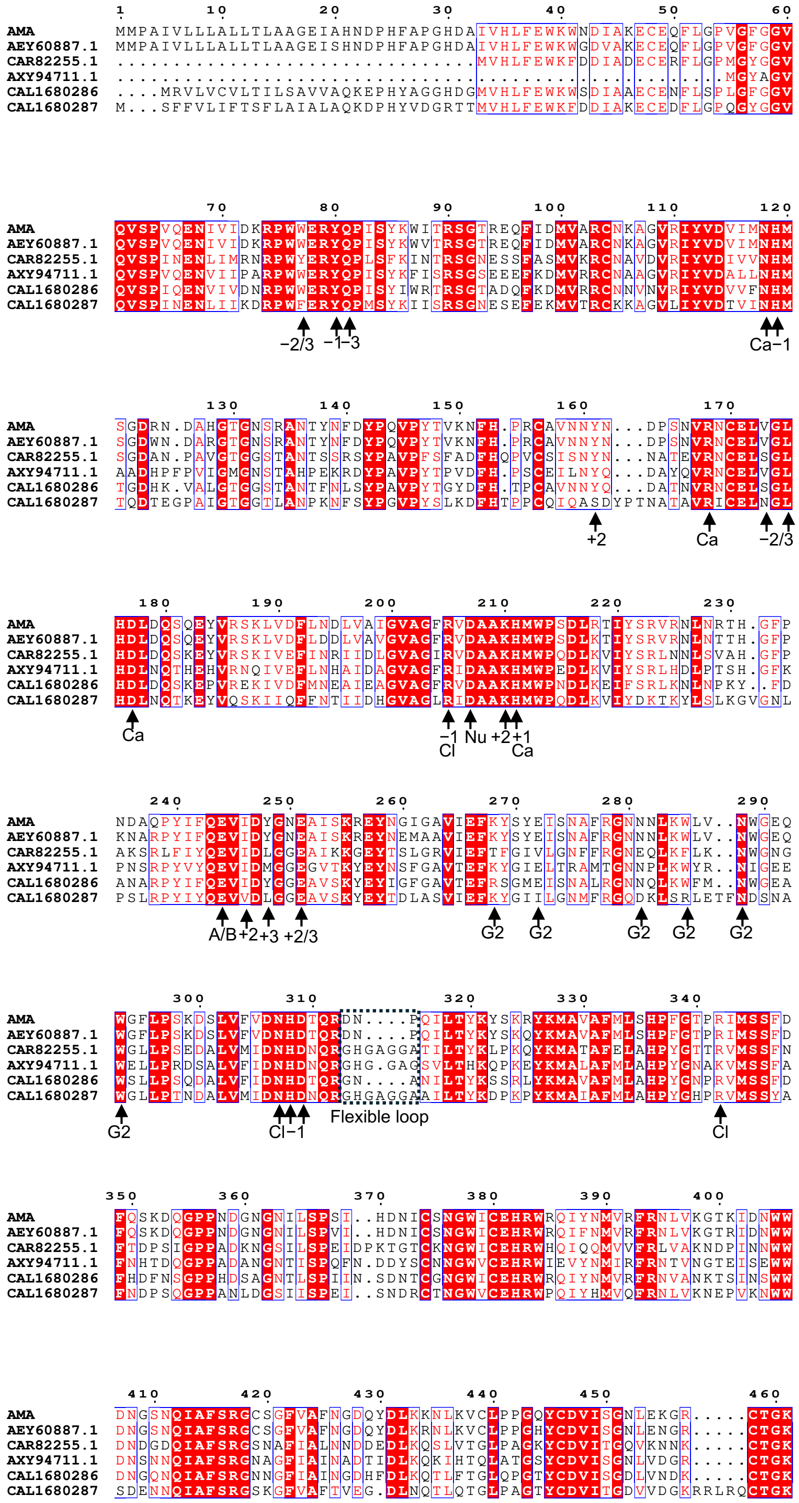

Figure S2. Multiple sequence alignment of Hymenoptera putative α-amylases. AEY60887.1, *Apis cerana*; CAR82255.1, *Cotesia congregata*; AXY94711.1, *Habrobracon hebetor*; CAL1680286.1 and CAL1680287.1, *Lasius platythorax*. Multiple-sequence alignment was constructed as Figure 9.

(a)

GAAATAGCACATAATGATCCCCACTTTGCTCCGGGTCACGACGCGATTGTTCACCTGTTTGAATGGAAAT
GGAATGACATCGCCAAGGAGTGTGAACAGTTTCTGGGTCCGGTGGGCTTCGGCGGTGTGCAGGTTAGCCC
GGTTCAAGAGAATATTGTTATCGACAAGCGCCCATGGTGGGAACGTTATCAGCCGATTTCCTACAAGTGG
ATCACCCGTTCCGGTACTCGTGAACAGTTTATTGACATGGTGGCCCGTTGCAACAAAGCGGGTGTACGCA
TCTACGTGGACGTTATCATGAATCACATGAGCGGTGATCGTAACGATGCGCATGGTACGGGTAACAGCCG
CGCAAATACCTACAACTTCGATTACCCGCAGGTCCCGTATACCGTGAAGAATTTTCATCCGCGTTGCGCT
GTGAATAACTATAACGACCCGTCAAACGTTAGAAACTGCGAGCTGGTGGGGCTGCATGACCTGGATCAAA
GCCAGGAGTATGTTAGGTCCAAGCTGGTCGATTTCTTGAATGACTTGGTTGCTATCGGCGTTGCTGGTTT
CCGCGTGGATGCCGCAAAACATATGTGGCCGAGCGATTTACGTACCATTTACAGCCGCGTGCGGAACCTT
AATCGTACCCACGGCTTTCCGAATGATGCGCAGCCGTACATTTTTCAAGAGGTCATTGACTACGGCAACG
AAGCAATCAGCAAACGTGAATACAATGGTATTGGTGCGGTGATTGAATTTAAATATAGTTATGAGATCTC
TAATGCGTTCCGCGGTAACAACAATTTGAAGTGGCTGGTCAACTGGGGTGAGCAATGGGGATTCCTGCCT
TCTAAGGACAGCCTGGTATTTGTGGACAACCACGATACCCAGCGTGATAATCCGCAAATCTTGACCTACA
AGTATAGTAAGCGCTATAAGATGGCTGTCGCGTTCATGCTGTCCCATCCGTTTGGCACGCCGCGTATTAT
GAGCAGCTTTGACTTCCAATCTAAGGATCAAGGTCCGCCAAACGACGGCAATGGCAATATCCTGTCCCCG
TCGATTCACGACAACATCTGCTCCAACGGCTGGATTTGCGAGCACCGTTGGCGTCAGATCTACAACATGG
TTAGATTCCGCAACCTAGTGAAGGGTACGAAAATCGACAACTGGTGGGATAACGGCAGCAACCAGATAGC
GTTCAGCCGTGGTTGCAGCGGCTTCGTCGCGTTCAACGGCGACCAATATGATCTCAAAAAAAACCTGAAA
GTTTGTCTGCCGCCAGGTCAGTACTGCGATGTAATCTCTGGCAACTTGGAAAAAGGTCGTTGTACCGGCA
AAATCGTGACCGTTGGCTCGGACGGCAACGCCAATATCGAGATCGGTGCAGGTGAGGAAGATGGTGTTCT
GGCAATCCATGTTAAGGCGAAAATGGCG

(b)

GAGATTGCACATAATGATCCTCACTTTGCTCCTGGACATGATGCTATTGTTCACCTTTTTGAATGGAAAT
GGAATGACATCGCTAAAGAGTGTGAACAATTCTTGGGTCCAGTTGGTTTCGGTGGTGTTCAAGTTTCTCC
TGTTCAAGAGAACATTGTTATTGATAAGAGACCATGGTGGGAAAGATACCAACCAATTTCTTACAAGTGG
ATTACTAGATCTGGTACTAGAGAGCAATTCATTGATATGGTTGCTAGATGTAACAAGGCTGGTGTTAGAA
TTTACGTTGATGTTATTATGAACCACATGTCTGGTGACAGAAATGATGCTCACGGTACTGGTAATTCTAG
AGCTAACACTTACAATTTCGATTACCCTCAAGTTCCATACACTGTTAAGAACTTTCACCCAAGATGTGCT
GTTAACAATTACAACGATCCATCTAATGTTAGAAACTGTGAATTGGTTGGTTTGCACGATTTGGATCAAT
CTCAAGAATACGTTAGATCTAAGTTGGTTGATTTCTTGAACGATTTGGTTGCTATTGGTGTTGCTGGTTT
CAGAGTTGATGCTGCTAAGCACATGTGGCCATCTGATTTGAGAACTATTTACTCTAGAGTTAGAAATTTG
AACAGAACTCACGGTTTTCCAAACGATGCTCAACCATACATTTTCCAAGAGGTTATTGATTACGGTAATG
AGGCTATTTCTAAGAGAGAGTACAATGGTATTGGTGCTGTTATTGAGTTCAAGTACTCTTACGAGATTTC
TAACGCTTTCAGAGGTAATAACAACTTGAAGTGGTTGGTTAATTGGGGTGAACAATGGGGTTTCTTGCCA

TCTAAGGATTCTTTGGTTTTCGTTGATAACCACGATACTCAAAGAGATAACCCACAAATTTTGACTTATA
AGTACTCTAAGAGATACAAGATGGCTGTTGCTTTCATGTTGTCTCATCCATTTGGTACTCCAAGAATTAT
GTCTTCTTTTGATTTCCAATCTAAGGATCAAGGTCCTCCAAATGATGGTAACGGTAATATTTTGTCTCCA
TCTATTCACGATAACATTTGTTCTAATGGTTGGATTTGTGAGCACAGATGGAGACAAATTTACAACATGG
TTAGATTCAGAAACTTGGTTAAGGGTACTAAGATTGATAACTGGTGGGATAACGGTTCTAACCAAATTGC
TTTTTCTAGAGGTTGTTCTGGTTTCGTTGCTTTCAACGGTGACCAATACGATTTGAAAAAGAATTTGAAG
GTTTGTTTGCCACCTGGTCAATACTGTGATGTTATTTCTGGTAACTTGGAGAAGGGTAGATGTACTGGTA
AAATTGTTACTGTTGGTTCTGATGGTAACGCTAATATTGAAATCGGTGCTGGAGAAGAAGATGGTGTTTT
GGCTATTCATGTCAAGGCTAAGATGGCT

Figure S3. Synthesized sequence of the AMA gene. (a) for *E. coli* expression. (b) for *P. pastoris* expression.

Table S1. Sequences of primers for site-directed mutagenesis.

Primer	Sequence (5′→3′)
W77A_s	CCATGGGCGGAAAGATACCAACCAATT
W77A_a	TCTTTCCGCCCATGGTCTCTTATCAAT
Q81A_s	AGATACGCACCAATTTCTTACAAGTGG
Q81A_a	AATTGGTGCGTATCTTTCCCACCATGG
Y161A_s	AACAATGCCAACGATCCATCTAATGTT
Y161A_a	ATCGTTGGCATTGTTAACAGCACATCT
V173A_s	GAATTG**GCT**GGTTTGCACGATTTGGATCA
V173A_a	CAAACCAGCCAATTCACAGTTTCTAACAT
L175A_s	GTTGGTGCGCACGATTTGGATCAATCT
L175A_a	ATCGTGCGCACCAACCAATTCACAGTT
K210A_s	GCTGCTGCGCACATGTGGCCATCTGAT
K210A_a	CATGTGCGCAGCAGCATCAACTCTGAA
I229A_s	GAGGTTGCTGATTACGGTAATGAGGCT
I229A_a	GTAATCAGCAACCTCTTGGAAAATGTA
E251A_s	GGTAATGCGGCTATTTCTAAGAGAGAG
E251A_a	AATAGCCGCATTACCGTAATCAATAAC
Y248A_s	ATTGATGCCGGTAATGAGGCTATTTCT
Y248A_a	ATTACCGGCATCAATAACCTCTTGGAA
K268A_s	GAGTTCGCGTACTCTTACGAGATTTCTAAC
K268A_a	AGAGTACGCGAACTCAATAACAGCACCA
E272A_s	TCTTACGCGATTTCTAACGCTTTCAGAGG
E272A_a	AGAAATCGCGTAAGAGTACTTGAACTCAA
N281A_s	GGTAATGCCAACTTGAAGTGGTTGGTTAA
N281A_a	CAAGTTGGCATTACCTCTGAAAGCGTTAG
W285A_s	TTGAAGGCGTTGGTTAATTGGGGTGAACA
W285A_a	AACCAACGCCTTCAAGTTGTTATTACCTC
N288A_s	TTGGTTGCTTGGGGTGAACAATGGGGTTT
N288A_a	ACCCCAAGCAACCAACCACTTCAAGTTGT
W293A_s	GAACAAGCGGGTTTCTTGCCATCTAAGGA
W293A_a	GAAACCCGCTTGTTCACCCCAATTAACCA
